# Establishment of a human coronavirus HKU1 infection evaluation system using apical-out airway organoids

**DOI:** 10.1099/jgv.0.002269

**Published:** 2026-05-14

**Authors:** Masatoshi Kakizaki, Satoko Sugimoto, Miyuki Kawase, Sakurako Norito, Hisao Okabe, Hayato Go, Koichi Hashimoto, Kazuya Shirato

**Affiliations:** 1 Department of Respiratory Viruses, National Institute of Infectious Diseases, Japan Institute for Health Security, 4-7-1 Gakuen, Musashimurayama, Tokyo 208-0011, Japan; 2 Department of Pediatrics, School of Medicine, Fukushima Medical University, 1st Hikarigaoka, Fukushima, Fukushima 960-1295, Japan

**Keywords:** air–liquid interface culture, apical-out airway organoids, cytopathic effect, human coronavirus HKU1 (HCoV-HKU1)

## Abstract

Human coronavirus HKU1 (HCoV-HKU1) is difficult to study because it does not propagate in standard cell lines. Consequently, research has relied on the air–liquid interface model, which is limited by long culture periods and the absence of cytopathic effects (CPEs). To address these challenges, we established an infection evaluation system using apical-out airway organoids (AOAOs). Derived from human bronchial epithelial cells, AOAOs supported efficient HCoV-HKU1 replication, and immunofluorescence revealed a preferential infection of ciliated cells. Notably, HCoV-HKU1 induced a distinct CPE characterized by organoid dissociation, enabling the quantification of viral titres via the TCID_50_ assay. Applying this model to antiviral testing, the serine protease inhibitor nafamostat effectively inhibited viral infection and CPE, consistent with the known entry mechanism. Thus, AOAOs provide a rapid, scalable and reproducible platform for HCoV-HKU1 research, overcoming limitations of traditional systems. This approach enables reliable viral replication analysis, titre determination and drug evaluation and may accelerate research on difficult-to-culture respiratory viruses.

## Introduction

Human coronavirus HKU1 (HCoV-HKU1), which belongs to the genus *Betacoronavirus*, is one of the causative agents of the common cold. In 2005, it was first identified in an elderly patient with severe pneumonia in Hong Kong [1]. A major challenge in studying HCoV-HKU1 has been its inability to propagate in the standard cell lines commonly used in virological research [1]. Therefore, the isolation and propagation of this virus necessitate the use of an *in vitro* airway epithelial model, specifically an air-liquid interface (ALI) culture of human bronchial/tracheal epithelial cells (HBTECs) [2,4]. This HBTEC-ALI system faithfully reproduces the physiological environment of the human airway epithelium, making it a highly useful model for viral infection and replication [5]. However, this system has significant limitations for research, including a lengthy culture period and the difficulty in measuring viral titres due to the lack of a discernible cytopathic effect (CPE) upon infection.

To overcome these limitations, apical-out airway organoids (AOAOs) have recently been developed as a more tractable culture platform [6,8]. In AOAOs, the apical surface is exposed to the culture medium, which facilitates pathogen access. AOAOs also offer a significant time advantage, as they can be differentiated in approximately half the time required for ALI cultures. While several AOAO formation methods exist, the technique reported by [6] is particularly suited for large-scale production because it is performed in an extracellular matrix-free environment [5]. Importantly, AOAOs have been shown to support the replication of major respiratory viruses, such as respiratory syncytial virus, influenza virus and rhinovirus [6,9]. Furthermore, infection with these viruses induces a clear CPE, characterized by organoid dissociation, offering a potential means of viral quantification [6].

Given these advantages, we hypothesized that AOAOs could serve as a superior platform for the study of HCoV-HKU1. In this study, we sought to establish and validate an HCoV-HKU1 infection evaluation system using AOAOs, thereby addressing the key challenges associated with the traditional ALI model.

## Methods

### Generation of AOAOs

AOAOs were generated according to the manufacturer’s protocol, with the exception of the cell origin [10]. Briefly, HBTECs (Cat# FC-0035, Lifeline Cell Technology, Frederick, MD, USA) were cultured in a 1 : 1 mixture of PneumaCult-EX and PneumaCult-EX Plus media (Cat# 05008 and 05040, STEMCELL Technologies, Vancouver, Canada) using a T-75 Flask (Cat# 430641, Corning, One Riverfront Plaza, NY, USA). When HBTECs reached semi-confluence, ~120,000 cells in 1 ml of PneumaCult AOAO medium (Cat# 100-0620, STEMCELL Technologies) were added to 24-well AggreWell plates (Cat# 34415, STEMCELL Technologies) pre-coated with an anti-adherence rinsing solution (Cat# 07010, STEMCELL Technologies). Over the next 3–5 days, the cells aggregated into small clumps. Subsequently, the three-dimensional structures were transferred to flat-bottom 24-well plates pre-coated with an anti-adherence rinsing solution and further differentiated for 7–14 days, with half of the medium changed every 2–3 days. Terminally differentiated AOAOs were filtered through a 30-µm reversible strainer (Cat# 43-500030-03, pluriSelect, Leipzig, Germany) and seeded into 96-well flat-bottom plates at a density of ~100 AOAOs per well in 200 µl of AOAO medium (without heparin) for subsequent assays. The culture temperature was 37 °C throughout all stages.

### Quantification of differentiation markers by real-time qPCR

Total RNA was isolated from AOAOs using ISOGEN reagent (Cat# 311-02501, Nippon Gene, Tokyo, Japan). Reverse transcription to cDNA was performed using the High-Capacity cDNA Reverse Transcription Kit (Cat# 4368814, Applied Biosystems, Foster City, CA). Real-time qPCR was performed using the THUNDERBIRD Next SYBR qPCR Mix (Cat# QPX-201, TOYOBO CO., Ltd, Osaka, Japan) and a LightCycler 480 instrument (Roche, Basel, Switzerland) with the primer pairs shown in Table 1. The thermal cycling conditions were as follows: initial denaturation at 95 °C for 1 min, followed by 40 cycles of 95 °C for 15 s, 60 °C for 15 s and 72 °C for 30 s.

**Table 1.** Primers and probes

Target	Primer/probe	Sequence
GAPDH	Forward	AGAACATCATCCCTGCCTCTACTG
	Reverse	CCTCCGACGCCTGCTTCAC
FoxJ1	Forward	TCGTATGCCACGCTCATCTG
	Reverse	CGGATTGAATTCTGCCAGGT
*β*-Tubulin IV	Forward	AGATCGTGCACCTGCAGG
	Reverse	CATGGTATGTGCCTGTGG
MUC5AC	Forward	TACTCCACAGACTGCACCAACTG
	Reverse	CGTGTATTGCTTCCCGTCAA
KRT5	Forward	GAGGAATGCAGACTCAGTGGA
	Reverse	TAGCTTCCACTGCTACCTCCG
p63	Forward	CAGACTCAATTTAGTGAGCC
	Reverse	CTGCTGGTCCATGCTGTT
HCoV-HKU1 (nucleocapsid gene)	Forward	GTTGCTAATCACCAAGCTGACAC
	Reverse	CGTACCAGGCGGAAACCTAG
	Probe	(FAM) CCCTCCGATGTTTCGTCAAGGGATCCT (BHQ1)

BHQ, black hole quencher; FAM, fluorescein amidite.

### Virus and infection of AOAOs

Two clinical isolates of HCoV-HKU1 (Genotype A: Fukushima_OH774_2025, LC889301.1, Genotype B: Fukushima H815_2020, LC654447) [11], which were initially isolated using an HBTEC-ALI culture, were used for infection experiments. After RNase (Cat# 312-01931, Nippon Gene) treatment of the viral stock, the viral RNA copy numbers of the stock were calculated from standard curves obtained with the aid of control RNA templates. To evaluate the susceptibility of AOAOs to HCoV-HKU1 infection, the organoids were infected with HCoV-HKU1 at a dose of 1.0×10⁶ copies/well. The infected organoids were incubated for 2 h at 37 °C and 5% CO_2_. To remove unbound viral particles, the organoids were transferred to a 1.5 ml tube, centrifuged at 300 ***g*** for 5 min and washed twice with DMEM/F12 (Cat# 048-29785, FUJIFILM Wako Pure Chemical Corporation, OSAKA, Japan). The organoid pellet was then resuspended in PneumaCult AOAO medium, seeded into 96-well flat-bottom plates and cultured at 37 °C.

### Quantification of viral RNA by real-time reverse transcriptase-quantitative PCR

The culture supernatant was harvested at the indicated time points. Viral RNA was isolated using ISOGEN-LS reagent (Cat# 311-02621, Nippon Gene). Real-time reverse transcriptase quantitative PCR (RT-qPCR) was performed to detect HCoV-HKU1 RNA using the Fast Virus 1-step Master Mix (Cat# 4444432, Applied Biosystems) and a LightCycler 480 instrument with the primers and probe listed in Table 1. Control RNAs of nucleoprotein (N) gene were synthesized using the sequences of clinical isolates (GenBank accession no. LC315650.2). The template for RNA transcription was prepared by reverse transcriptase PCR (RT-PCR) amplification using the following primers: forward, 5′-TAATACGACTCACTATAGGGATATTGGTATAAACACAACC-3′, and reverse, 5′-TATCAGGTTTCACTATAGAATCAG-3′. The forward primer contained a T7 promoter sequence at the 5′-end. After purification of the PCR amplicons, control RNAs were synthesized with the MEGAscript T7 Kit (Cat# AM1333, Thermo Fisher Scientific, Waltham, MA) according to the manufacturer’s instructions. The copy number of synthesized RNA (398 nt) was calculated based on the molecular weight and concentration determined by the QuantiFluor RNA system and Quantus Fluorometer (Cat# E6150, Promega, Fitchburg, WI). The copy number-adjusted control RNA was diluted with PCR-grade water containing 10 µg ml^−1^ of yeast RNA (R6750, Sigma, St. Louis, MO) for storage at −80 °C. RNA copy numbers in samples were calculated from standard curves generated using serial dilutions of the control RNA templates. The thermal cycling conditions were as follows: reverse transcription at 50 °C for 5 min and RT inactivation/initial denaturation at 95 °C for 20 s, followed by 45 cycles of 95 °C for 15 s, 60 °C for 30 s and 72 °C for 30 s.

### Virus titration

The infectivity titres of the stocked HCoV-HKU1 were analysed using AOAOs in PneumaCult AOAO medium. CPE, characterized by organoid dissociation, was examined using a BZ-X810 microscope (KEYENCE, Osaka, Japan). Viral infectivity titres were expressed as the TCID_50_ per millilitre, calculated according to the Behrens–Kärber method.

### Antiviral testing

To test for antiviral effects on HCoV-HKU1 replication, 10 µM (2S,3S)-trans-epoxysuccinyl-l-leucylamido-3-methylbutane ethyl ester (EST) (Cat# 330005, Calbiochem, San Diego, CA, USA) or 10 µM nafamostat (Cat# N0959, Tokyo Chemical Industry, Tokyo, Japan) was added to the medium after reconstitution in DMSO (Cat # D2650, Sigma-Aldrich). At the designated time points, 10 µl of the culture medium supernatant was harvested for the detection of the viral load.

### Immunofluorescence staining

AOAOs were fixed with 4% paraformaldehyde for 20 min and washed twice with PBS. The organoids were permeabilized with 1% Triton X-100 for 15 min and then blocked with 3% BSA in PBS containing 0.1% Tween 20 and 0.2% Triton X-100 (PBSTT) for 1 h. Primary antibodies were diluted in PBSTT and incubated with the organoids overnight at room temperature in a tube with gentle agitation. AOAOs were stained for the epithelial markers acetylated *α*-tubulin (Cat # CL594-66200, Proteintech, Rosemont, IL, USA) and MUC5AC (Cat # 61193, Cell Signaling Technology, Danvers, MA, USA) and for the HCoV-HKU1 spike protein (Cat # MAB10936, R and D SYSTEMS, McKinley, NE, USA). The AOAOs were then washed three times with PBSTT, and the respective secondary antibodies were incubated at room temperature in a tube with gentle agitation for 1 h. The following secondary antibodies were used: CoraLite488-conjugated Donkey Anti-Mouse IgG (H+L) (Cat # SA00013-5, Proteintech), CoraLite488-conjugated Donkey Anti-Rabbit IgG (H+L) (Cat # SA00013-6, Proteintech) and CoraLite594-conjugated Donkey Anti-Rabbit IgG (H+L) (Cat # SA00013-8, Proteintech). Nuclei were visualized with DAPI (Cat # 19178-91, Nacalai). Stained AOAOs were mounted with ProLong Diamond Antifade Mountant (Cat # P36961, Thermo Fisher Scientific) and examined using a confocal laser scanning FV-3000 microscope (Olympus, Tokyo, Japan).

### Statistical analyses

Statistical analyses were performed using GraphPad Prism 9 software (version 9.4.1). Statistical analyses included Student’s *t*-tests and two-way ANOVA followed by Tukey’s post hoc test for multiple comparisons. Values of *P*<0.05 were considered significant. Data are presented as the mean±sd.

## Results

**Fig. 1.**
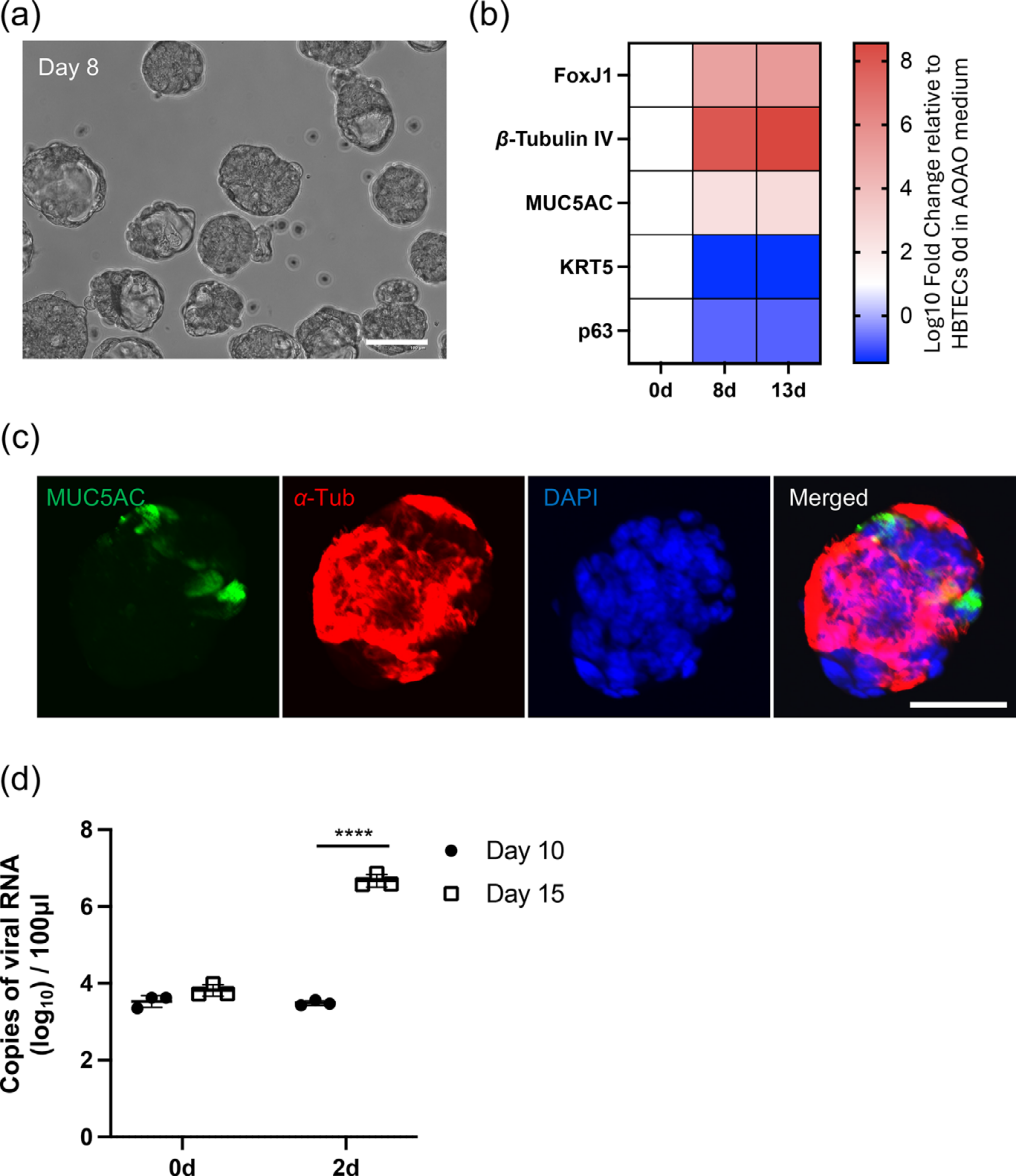
Generation of AOAOs. (**a**) Bright-field image of HBTECs cultured in PneumaCult AOAO medium at day 8. White bar indicates 100 µm. (**b**) Heatmap of differentiation marker expression levels. (**c**) Immunofluorescence of AOAOs at day 13. AOAOs were stained to detect acetylated *α*-tubulin (red), MUC5AC (green) and nuclei using DAPI (blue). White bar indicates 50 µm. (**d**) Day 10 AOAOs and day 15 AOAO were infected with HCoV-HKU1 (Genotype B) at a dose of 1.0×10⁶ copies/well. At 2s day p.i., viral RNAs in the culture supernatant were detected by real-time RT-PCR. Error bars indicate the sd of triplicate well. Mean values±sd are shown. Statistical significance was determined with Student’s t-test, ****P<0.0001.

HBTECs cultured in PneumaCult AOAO medium formed organoid-like structures by day 8 (Fig. 1a), accompanied by increased gene expression of ciliated and goblet cell differentiation markers (Fig. 1b). The gene expression levels of these differentiation markers remained unchanged between day 8 and day 13 of culture (Fig. 1b). Immunofluorescent analysis revealed that acetylated-*α*-tubulin (a ciliated cell marker) and MUC5AC (a goblet cell marker) were expressed, with a higher proportion of ciliated cells than goblet cells (Fig. 1c). This finding is consistent with the gene expression data. Next, to determine whether culture duration affects susceptibility to HCoV-HKU1, we inoculated HCoV-HKU1 into AOAOs at 10 and 15 days post-culture. Although the organoids appeared well-differentiated by day 8, their susceptibility to the virus was significantly lower on day 10 than on day 15 (Fig. 1d). This result indicates that the host environment required for efficient viral infection or replication was not fully established until day 15 of culture.

**Fig. 2.**
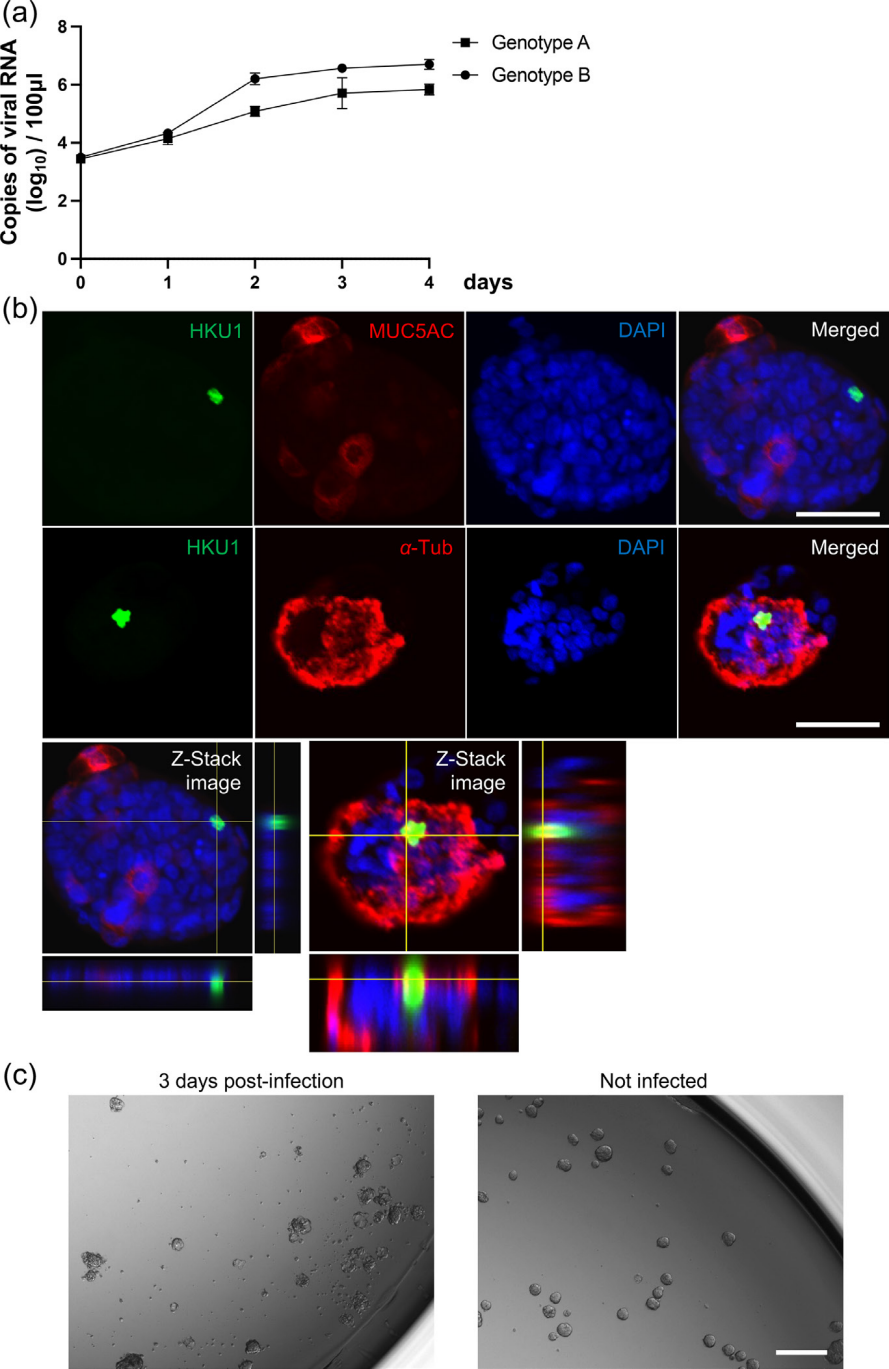
Replication ability and CPE after HCoV-HKU1 infection in AOAOs. (**a**) Replication kinetics in AOAOs infected with HCoV-HKU1 (Genotypes A and B) at a dose of 1.0×10⁶ copies/well. Viral RNAs in the culture supernatant were detected by real-time RT-PCR at 0, 1, 2, 3, 4 days p.i. (**b**) Immunofluorescence of AOAOs infected with HCoV-HKU1 (Genotype B) at 1 or 2 days p.i. AOAOs were stained to detect acetylated *α*-tubulin (red), MUC5AC (red), HCoV-HKU1 spike protein (green) and nuclei using DAPI (blue). White bar indicates 50 µm. (**c**) Bright-field image of AOAOs infected with HCoV-HKU1 (Genotype B) at 3 days p.i. White bar indicates 200 µm.

Serial measurements of the viral load following HCoV-HKU1 (Genotypes A and B) infection revealed that it peaked on days 2–3 post-infection, after which it plateaued (Fig. 2a). Furthermore, HCoV-HKU1 could be replicated in AOAOs regardless of genotype. Subsequently, immunofluorescence and Z-Stack imaging demonstrated that HCoV-HKU1 infects ciliated cells, not goblet cells (Fig. 2b). This finding is consistent with previous reports based on analyses using HBTEC-ALI [2]. Next, to establish a titration method for the HCoV-HKU1 stock using AOAOs, we determined the time point at which organoid dissociation occurred post-infection. Dissociation was observed at 3 days post-infection (Fig. 2c). Based on this observation, we measured the viral titre at 3 days post-infection (Table 2). The TCID_50_/ml, calculated using the Behrens–Kärber method, was considerably lower than the viral copy count determined by real-time RT-qPCR (Table 2).

**Table 2.** Viral titration

Dilution ratio	Column 1	Column 2	Column 3	TCID_50_/ml	Viral copies/ml of HKU1 stock
10^−2^	○	○	○	3.2×10^5^	1.2×10^9^
10^−3^	○	○	○		
10^−4^	○	○	○		
10^−5^	×	×	×		
10^−6^	×	×	×		
10^−7^	×	×	×		
10^−8^	×	×	×		
10^−9^	×	×	×		

○, CPE detected; ×, CPE not detected.

**Fig. 3.**
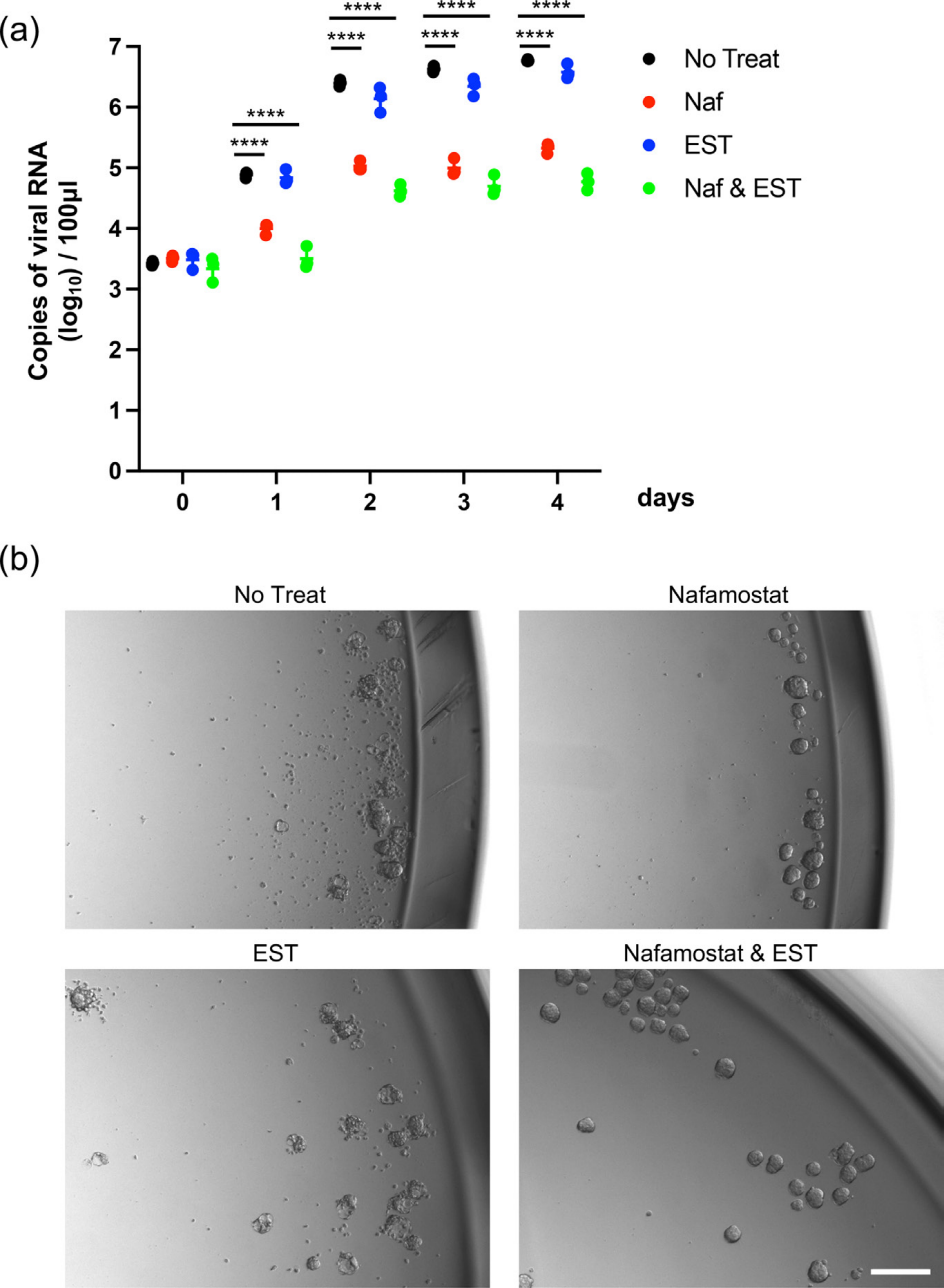
Antiviral test. (**a**) AOAOs were infected with the virus (Genotype B) at a dose of 1.0×10⁶ copies/well in the absence (NT) or presence of either nafamostat (Naf) or EST or both (Naf and EST). At 0, 1, 2, 3 and 4 d.p.i., viral RNAs in the culture supernatant were detected by real-time RT-PCR. The y-axis shows the viral RNA copies (log_10_)/100 µl. Statistical significance was determined with two-way ANOVA. Error bars indicate the sd of triplicate well. Mean values±sd are shown. Multiple comparisons among different groups were adjusted with Tukey’s multiple comparison test, ****P<0.0001. (**b**) Bright-field image of AOAOs infected with HCoV-HKU1 (Genotype B) at a dose of 1.0×10⁶ copies/well in the absence or presence of either nafamostat or EST or both at 3 d.p.i. White bar indicates 200 µm.

AOAOs can be generated on a large scale, making them a valuable tool for evaluating antiviral drug efficacy. Therefore, to assess whether AOAOs can be used for antiviral drug evaluation, we examined the effects of the serine protease inhibitor nafamostat and the cathepsin L inhibitor EST on the viral load and CPE. HCoVs enter cells via two distinct pathways: the endosomal pathway using cathepsins to activate spike protein and the cell-surface or early endosome pathway using extracellular proteases such as transmembrane protease serine 2 (TMPRSS2) [4,12,14]. HCoV-HKU1 was inhibited by nafamostat by ~1.5 log_10_ at 4 days post-infection, whereas EST had no effect (Fig. 3a). In addition to viral copy number, we additionally measured the viral titre at 4 days post-infection under drug treatment (Table 3). This data supported the real-time RT-qPCR results. This result is consistent with previous studies using HBTEC-ALI [4] and indicates that HCoV-HKU1 primarily uses the cell-surface pathway for cell entry into AOAOs. Furthermore, nafamostat treatment prevented the development of CPE (Fig. 3b). The CPE observed in AOAOs is highly distinctive, making them an excellent model for evaluating antiviral drugs.

**Table 3.** Viral titration after drug treatments

Treatment	TCID_50_/ml	Viral copies/ml
No treatment	3.2×10^2^	4.13×10^7^
Nafamostat	3.2×10^1^	7.45×10^5^
EST	3.2×10^2^	8.35×10^6^
Nafamostat and EST	N.D	3.06×10^5^

N.D, not detected.

## Discussion

This study establishes an AOAO-based evaluation system for HCoV-HKU1 infection, presenting an experimental platform that complements the conventional HBTEC-ALI system, whose limitations include protracted differentiation, lack of CPE and difficulty in titre measurement. By demonstrating reproducible HCoV-HKU1 replication, a visible dissociation-type CPE and antiviral sensitivity readouts in AOAOs, we extend the utility of this platform from RSV, influenza and rhinovirus to coronaviruses [6,9], representing a novel contribution to the field.

As HCoV-HKU1 is notoriously difficult to propagate in standard immortalized cell lines, the HBTEC-ALI system has long been an essential tool. Foundational HBTEC-ALI studies demonstrated HCoV-HKU1 replication in ciliated epithelia and enabled clinical isolate characterization [2,3]. Moreover, the HBTEC-ALI model facilitates long-term experiments, allowing for observation of the prolonged effects of viral infection on the host and virus [15]. However, its preparation typically requires 4–6 weeks and is not readily scalable for higher-throughput assays. In contrast, the AOAO system offers a shorter turnaround time, scalability and a quantifiable CPE, thereby providing a more tractable infection evaluation platform that complements the ALI system. AOAOs and HBTEC-ALI possess distinct strengths; selecting the appropriate model based on research objectives will accelerate future advances in virology.

Stroulios *et al*. previously reported that apical-out polarity of AOAOs confers susceptibility to IAV/IBV/RV-A16 and produces a clear dissociation-type CPE, enabling antiviral evaluation [6]. Building on this, we leveraged HKU1-induced organoid dissociation as a quantitative endpoint to establish TCID_50_-based titration. However, there is a discrepancy between the TCID_50_ titre and viral copy numbers quantified by real-time RT-qPCR. Real-time RT-qPCR detects non-infectious viral particles with structural defects or genomic deletions, as well as RNA released from dead cells. Furthermore, the virus used in this study was propagated in an ALI culture system. Since the ALI culture system is rich in mucus, the stock virus solution also contains abundant mucus, suggesting that mucus may be inhibiting infection. Recent work has also demonstrated the suitability of AOAOs for higher-throughput neutralization testing with viruses like RSV [9]. Our findings align with this trajectory, further validating AOAOs as an emerging platform for functional screening assay.

Our data reveal a critical temporal component in viral susceptibility. Although AOAOs appear morphologically differentiated by day 8, their susceptibility to HCoV-HKU1 infection was significantly higher on day 15 compared to day 10. This result suggests that full functional maturity – likely involving the upregulation of host factors essential for HCoV-HKU1 entry, such as the glycan receptor sialoglycan and the type II transmembrane serine protease TMPRSS2 [16,19] – follows initial morphological development. This finding underscores the importance for researchers using organoid models to characterize the specific temporal dynamics of susceptibility for their pathogen of interest, rather than relying solely on visual markers of differentiation.

Crucially, the AOAO model recapitulates the known *in vivo* cellular tropism of HCoV-HKU1 [2,3]. Our immunofluorescence analysis confirmed that HCoV-HKU1 specifically infects ciliated cells (acetylated *α*-tubulin^+^) and not goblet cells (MUC5AC^+^). Furthermore, protease inhibitor experiments revealed that the entry mechanism of HCoV-HKU1 in AOAOs is consistent with previous findings in the HBTEC-ALI system [4,20]. These results validate the AOAO system as a high-fidelity model for studying authentic virus–host interactions at the cellular level.

A limitation of this study is that comparison with HBTEC-ALI culture has not been performed. Head-to-head comparisons with matched HBTEC-ALI cultures, assessing metrics such as virus release kinetics and non-immune host responses, would further clarify the concordance and complementarity of the two models. Finally, implementing automated image-based scoring of the CPE and ciliary motion analysis would enhance both objectivity and throughput.

In conclusion, the AOAO platform provides (i) direct apical access for infection, (ii) multilayered readouts (CPE, RT-qPCR and immunostaining) and (iii) reproducible protease-inhibitor sensitivity profiles, all within a short and scalable workflow. This system complements the HBTEC-ALI model by shortening turnaround time and being compatible with scaled microwell formats, facilitating medium-throughput studies of viral entry, replication and pharmacologic inhibition for HCoV-HKU1 and other difficult-to-culture respiratory viruses.

## Abbreviations

ALI: air–liquid interface
AOAO: apical-out airway organoid
CPE: cytopathic effects
HBTEC: human bronchial/tracheal epithelial cell
HCoV-HKU1: human coronavirus HKU1
PBSTT: PBS containing 0.1% Tween 20 and 0.2% Triton X-100
RT-PCR: reverse transcriptase PCR
RT-qPCR: reverse transcriptase quantitative PCR
TMPRSS2: transmembrane protease serine 2
